# Are We Ready for the Arrival of the New COVID-19 Vaccinations? Great Promises and Unknown Challenges Still to Come

**DOI:** 10.3390/vaccines9020173

**Published:** 2021-02-18

**Authors:** Davide Gori, Chiara Reno, Daniel Remondini, Francesco Durazzi, Maria Pia Fantini

**Affiliations:** 1 Department of Biomedical and Neuromotor Sciences, University of Bologna, 40138 Bologna, Italy; davide.gori4@unibo.it (D.G.); mariapia.fantini@unibo.it (M.P.F.); 2 Department of Physics and Astronomy, University of Bologna, 40138 Bologna, Italy; daniel.remondini@unibo.it (D.R.); francesco.durazzi2@unibo.it (F.D.)

**Keywords:** SARS-CoV-2, COVID-19, vaccination, vaccine hesitancy, social media, Twitter

## Abstract

While the SARS-CoV-2 pandemic continues to strike and collect its death toll throughout the globe, as of 31 January 2021, the vaccine candidates worldwide were 292, of which 70 were in clinical testing. Several vaccines have been approved worldwide, and in particular, three have been so far authorized for use in the EU. Vaccination can be, in fact, an efficient way to mitigate the devastating effect of the pandemic and offer protection to some vulnerable strata of the population (i.e., the elderly) and reduce the social and economic burden of the current crisis. Regardless, a question is still open: after vaccination availability for the public, will vaccination campaigns be effective in reaching all the strata and a sufficient number of people in order to guarantee herd immunity? In other words: after we have it, will we be able to use it? Following the trends in vaccine hesitancy in recent years, there is a growing distrust of COVID-19 vaccinations. In addition, the online context and competition between pro- and anti-vaxxers show a trend in which anti-vaccination movements tend to capture the attention of those who are hesitant. Describing this context and analyzing its possible causes, what interventions or strategies could be effective to reduce COVID-19 vaccine hesitancy? Will social media trend analysis be helpful in trying to solve this complex issue? Are there perspectives for an efficient implementation of COVID-19 vaccination coverage as well as for all the other vaccinations?

## 1. Introduction

On 27 January 2020, vaccination campaigns against severe acute respiratory syndrome coronavirus 2 (Sars-CoV-2) officially started across Europe. Symbolically, this day represents a great achievement for science occurring approximately one year after the reporting to the World Health Organization (WHO) of a cluster of pneumonia of unknown origin in Wuhan City, Hubei Province of China [1]. The development and production of vaccines against SARS-CoV-2 took place at an impressive speed, considering, for example, that the first US trial began 66 days later the online publication of the viral RNA sequence [2]. A major innovation consists in the use of technologies, besides the more traditional ones, that have never been used in a licensed vaccine before, such as mRNA platforms [3]. While the SARS-CoV-2 pandemic continues to strike and collect its death toll throughout the globe, the availability of safe and effective vaccines can truly represent a precious and much-needed tool to mitigate its effect, together with non-pharmaceutical interventions, and, in particular, to offer early protection to some vulnerable strata of the population (i.e., the elderly). From a global perspective, in the long term, effective vaccine mass utilization will also be capable of reducing the social and economic burden of the pandemic and paving the way for an acceptable exit strategy from the current crisis.

When developing vaccination strategies, it is crucial to consider how the different segments of the population are affected by the infection. Older population groups have proved to be highly susceptible to this infection and to severe disease, while children seem to be less affected by the epidemic [4,5,6]. Nonetheless, young people, mainly asymptomatic or paucisymptomatic, may contribute to the spread of the infection, given their social habits and school attendance. Vaccination of older people is the optimal age-based strategy to reduce the burden of the disease and the strain on healthcare systems, while vaccination of younger adults could reduce the incidence of the infection.

Vaccination programs should optimize vaccine allocation maximizing public health benefit and taking into account that in the next months, the demand for vaccines will exceed supply [6,7]. Moreover, the rapid scale-up of vaccination campaigns, besides the basic key principle of equitable access, also requires considering several other aspects, such as the whole delivery infrastructure, logistic coordination and surveillance of adverse events after immunization [7].

Surely, as the success of the vaccination deployment greatly relies on the uptake by the targeted population, efforts in transparent and effective communication campaigns are of paramount importance to counteract misinformation and vaccine hesitancy and to build trust in the population to ensure that adequate vaccination coverage is reached [7].

Since the outbreak of the pandemic, we have been overwhelmed by information from different channels, from the more traditional ones to social media, and the same is happening with vaccines. These multiple sources of information have the great potential to influence public opinion, therefore having an impact on vaccination acceptance and eventually on vaccine hesitancy [8].

The impressive speed at which vaccines against SARS-CoV-2 have been developed and produced certainly represents a tremendous accomplishment for science, but at the same time involves several open issues that we can summarize as follows: (1) which type of vaccines are available and what we know so far about their efficacy and safety, and type of vaccination strategies; (2) what we know about acceptance of anti-COVID-19 vaccination; (3) what is the role of media in the information/communication campaign, focusing on vaccine hesitancy.

To answers these questions according to available knowledge, we conducted a narrative review. PubMed (http://www.ncbi.nlm.nih.gov/pubmed/), PROQUEST (https://search.proquest.com/) and Turning Research Into Practice (TRIP) (http://www.tripdatabase.com/) database were searched for studies published up to 30 January 2021 in order to retrieve the most relevant evidence on these topics. Studies potentially eligible according to the pertinence for the different topics previously described were identified from the abstracts; full texts of relevant studies were assessed for inclusion, and their reference lists were searched for additional studies. Inconsistency was resolved through consensus. Hand-searching techniques (i.e., “snowballing” or pursuing references of references) were also applied to find additional articles [9].

We also present a case study for Italy with preliminary data on the volume of social media in relation to relevant public communication about anti-COVID-19 vaccines.

## 2. Type of Anti-COVID-19 Vaccines, Their Efficacy and Safety, and Vaccination Strategies

The public health emergency has accelerated both the development and the approval of COVID-19 vaccines. The European Medicines Agency (EMA) put in place rapid review procedures and a dedicated expert task force to review applications as rapidly as possible while ensuring at the same time robust scientific opinions [10].

One year after the outbreak of the pandemic, as of 31 January 2021, the vaccine candidates worldwide are 292, of which 70 in clinical testing [11]. Three anti-COVID-19 vaccines have been authorized for use in the EU following recommendations by the EMA [12]: BioNTech-Pfizer (mRNA), Moderna (mRNA) and AstraZeneca (nonreplicative viral vector). Moreover, three contracts have been concluded to purchase a vaccine once proven safe and effective: Sanofi-GSK (protein subunit), Johnson and Johnson (nonreplicative viral vectorl) and CureVac (mRNA). In addition, the European Commission has concluded exploratory talks with the European companies Novavax (protein subunit) and Valneva (inactivated virus).

Besides the extraordinary timing that led to the development and authorization of vaccines, a point of great novelty is the use of technologies that have never been used in a licensed vaccine before [3], on which the three vaccines authorized for use in the EU are based. Vaccines by BioNTech-Pfizer and Moderna are mRNA-based, a type of technology long-studied and which has always been considered really promising [13,14]. AstraZeneca vaccine relies on a nonreplicative recombinant chimpanzee adenovirus-based platform. All these three vaccines require two doses while a point of difference is represented by storage conditions; the AstraZeneca vaccine does not require low-temperature storage, and this is a key element for logistical considerations when planning a mass vaccination campaign.

Both BioNTech-Pfizer and Moderna vaccines showed high efficacy in terms of reduction of symptomatic cases, 95% and 94.1%, respectively [15,16]. However, a major question that has not yet been answered remains, namely whether the vaccines protect against asymptomatic infection and transmission to susceptible people.

The AstraZeneca vaccine demonstrated around 60% efficacy in reducing the number of symptomatic COVID-19 cases in vaccinated people across four clinical studies involving around 24,000 individuals [17]. Of note, participants were between 18 and 55 years old. Therefore to date, it is not certain how well this vaccine could work in older participants. Based on these considerations, in Italy, the regulatory agency (AIFA) recommended this type of vaccine for people aged between 18 and 55, while mRNA vaccines should be prioritized for older and fragile people [18]. However, recently, WHO recommended this type of vaccine also for people aged 65 or older [19].

As regards safety, the reported side effects for all three vaccines were usually mild or moderate. Allergic reactions after the first dose of an mRNA vaccine have been reported, and scientists suspect that they may be due to a specific compound, polyethylene glycol (PEG), never used before in an approved vaccine, but further studies are necessary to confirm or refute this hypothesis [20]. Postmarketing studies are needed to monitor potential side effects in the long term and taking into account that several segments of the population were not recruited in efficacy and safety trials, such as pregnant women, severely immunocompromised individuals, children and adolescents.

Given the availability of these new vaccines, roughly a month since the official beginning of the vaccination campaign, two major issues are now emerging.

First, uncertainty on the number of doses and their arrival time already exists, leading to organizational problems and changes in the vaccine campaign strategic plans.

Second, new variants of the virus have been identified during the past months. The risk assessment by the European Centre for Disease Prevention and Control (ECDC) has been increased to high/very high in relation to the introduction and community spread of three new variants that have been identified in the UK, South Africa and Brazil [21]. Therefore, an open question that urgently needs an answer is whether these new variants will escape recognition by the immunity induced by vaccines [22]. What is known so far is that variant B.1.1.7. (the often so-called “UK strain”) has been neutralized in vitro by serum samples of recipients of the Pfizer-BioNTech and Moderna mRNA vaccines [22]. On the other side, variant B.1.351 (identified in South Africa) and the close relative identified in Brazil have more alarming sequence changes, thus raising serious concerns about vaccine efficacy [22]. However, the strong response stimulated by mRNA vaccines in terms of neutralizing antibodies could be enough to avoid a significant loss of effectiveness, while other types of vaccines that induce lower levels of these neutralizing antibodies could be less effective [22]. It is therefore highly important to put in place a sequencing surveillance system to monitor the emergence of new variants and to evaluate the effectiveness of vaccines thoroughly and continuously, trying in the meantime to reach adequate levels of vaccination as soon as possible. Of note, researchers are considering redesigning vaccines against SARS-CoV-2, and some coronavirus vaccine makers are already taking into account updating their vaccines to counter these new strains [23]. Thanks to validated platforms long studied before the pandemic, this process could take place relatively rapidly, potentially following the updated model of seasonal flu vaccines.

## 3. Acceptance towards Anti-COVID-19 Vaccination

Over the course of the pandemic, several studies have been conducted, and gray literature published to capture and address the attitude towards anti-COVID-19 vaccines. It has been immediately clear that the immense efforts to develop and produce vaccines against a new pathogen in a really short period of time could be undermined by refusal or hesitancy, with the concrete risk of making herd immunity difficult to reach.

In a study carried out in Italy early after the national lockdown (May 2020) on a random sample of 1004 adult citizen, 15% of respondents stated they would probably refuse the vaccine, while 26% declared to be hesitant [24]; moreover, health engagement (more responsible about one’s health; “health engaged people”) was found to be positively related to the intention to be vaccinated. No difference was found between genders, and a partial difference was found across age groups.

Only about one-half of American adults plan to perform anti-COVID-19 vaccination, as emerged from a survey of 1117 American adults last December. Only 3 out of 10 respondents were very or extremely confident in vaccine safety and effectiveness [25].

In a longitudinal study carried out between April and 8 December 2020, participants were asked how likely they were to get vaccinated for coronavirus once a vaccine was available. The self-reported likelihood of getting the COVID-19 vaccine declined from 74% in early April to 56% in early December 2020 [26]. In particular, during the last survey, lower levels were reported among women, Black individuals, people with lower education and younger, while higher levels were found among adults aged 65 years and older vs. those aged 18–49 years.

A general declining trend in the intention to be vaccinated against COVID-19 occurred globally. An online survey conducted in the period 8–13 October 2020, across 15 countries comprising more than 18,000 adults found that on average, 73% of respondents stated they would get vaccinated; three months earlier, the average was 77%. Of note, wide variations were reported across countries, with a sharp decline in China (85% versus 97%) and a less pronounced one in Italy (65% versus 67%) [27].

Further studies are necessary to understand the determinants of vaccine acceptance and vaccine hesitancy in the different geographical and cultural contexts in relation to the pandemic trends and external events. For example, a special focus should be devoted to vaccine safety since for the general population, and it is sometimes difficult to understand how science has achieved the production of effective vaccines in such a short time since it usually takes years of research and experiments to make a vaccine. In this case, it should be pointed out that the technology was developed and validated long before this pandemic for other epidemics, and it has just been effectively re-purposed in order to tackle the virus spread.

The World Health Organization included vaccine hesitancy in the list of the ten major threats to global health in 2019 [28]. The Strategic Advisory Group of Experts (SAGE) Working Group on Vaccine Hesitancy defines vaccine hesitancy as the “delay in acceptance or refusal of vaccination despite the availability of vaccination services”. Vaccine hesitancy is complex and context-specific, varying across time, place, and vaccines. It is influenced by factors such as complacency, convenience, and confidence [29], the so-called three Cs model.

In the “3 Cs” model, vaccination confidence is defined as trust in (i) the real effectiveness and safety of vaccines; (ii) the system that delivers them, including the reliability and competence of the health services, health stakeholders and health professionals and (iii) the motivations of policymakers who decide on the needed vaccines as well as the prioritization groups. Vaccination complacency exists where perceived risks of vaccine-preventable diseases are low and, hence, vaccination is deemed unnecessary. Complacency about a particular vaccine or about vaccination, in general, is influenced by many factors. A vaccination program success may, paradoxically, result in a reduction of complacency and, ultimately, hesitancy, as individuals weigh risks of vaccination with a particular vaccine against risks of the disease the vaccine prevents, which is no longer commonly seen in the community (i.e., poliomyelitis). Vaccination convenience is a significant factor when physical availability, affordability and willingness-to-pay, geographical accessibility, ability to understand and appeal of immunization services affect uptake. The real and/or perceived quality and the degree to which vaccination services are delivered at a time and place and in a cultural context that is convenient and comfortable also affect the decision to be vaccinated and could lead to vaccine hesitancy.

## 4. Role of Social Media in the Information/Communication Campaign, with a Particular Focus on Vaccine Hesitancy

Over the course of a pandemic, individuals received information from multiple sources, but the social media role is emerging [8]. A recent survey with Italian medical students highlighted that they rely mostly on official communication and websites, but they used social media as the principal source of information [30]. The content and spread of information and misinformation online may be a determinant of risk perception and vaccine hesitancy. In fact, some evidence underlined that social media contents might influence vaccine acceptance [31]. In a study on parents’ health beliefs, parents who were Facebook users appeared as being disproportionately oriented towards vaccine hesitancy because of emotional and narrative contents [32].

In a sentiment analysis carried out from June 2011 to April 2019 on 1,499,237 tweets to study the public perception regarding vaccination on Twitter, a growing general interest in vaccines over time has been found [8]. Most of the analyzed tweets were neutral, but there has been an increasing tendency of both negative and positive ones. Of note, positive tweets had higher engagement than the negative ones, and, consistent with other findings, neutral tweets had fewer retweets. Significant sentiment polarity variation was found depending on locations.

Other evidence showed that social media sites allow users from different countries to have public discussions about any topic, including vaccination, in real-time and with minor or absent filtering of correct information. Even worse, the online context and competition between pro- and anti-vaxxers show today a trend in which anti-vaccination movements tend to capture the attention of those who are hesitant even if they are numerically inferior to the followers of the pro-vaccine movement on the social networks [33].

Digital communities and networks could contribute to the increase in vaccine hesitancy since they allow the fast spread of rumors, fake news and myths regarding vaccination, along with the fact that people tend to follow people who agree with them [34]. It is possible, for example, to map the relationship between users with different opinions about vaccinations, the key leaders (“influencers”) over time [35]. It is hence of primary importance to monitor social media trends to measure how and how much vaccine hesitancy is spread in our communities.

## 5. A Case Study for Italy: A Twitter Volumes Analysis

**Figure 1:**
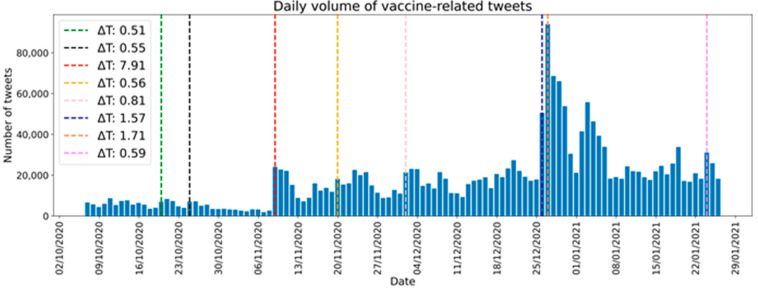
Daily volume of collected Italian tweets, from 7 October 2020 to 26 January 2020. Tweets were collected through several keywords: “vaccino”, “vaccini”, “vaccinazione”, “vaccinazioni”. A vertical line is drawn on the days where we observed a relative increase of tweets volume higher than 50%.

In Figure 1 and Table 1, we show an example for Italy, in which variations in the volume of tweets associated with COVID-19 (and thus indirectly quantifying the concern on the topic) seemed associated with some events that occurred in Italy in the same days driven by highly influential people. For this analysis, we defined a relative increase indicator as ΔT = (T_day_ − T_day-1_)/T_week_, where T is the volume of tweets on that day. Tweets were collected from 7 October 2020 to 26 January 2020 through several keywords. Vertical lines have been drawn on the days where we observed a relative increase of tweets volume higher than 50% corresponding to the first announcement by Pfizer of a new vaccine, a skeptical statement on the vaccine by a well-known physician and first doses of vaccine arrive in Italy and the first day of vaccination in Italy. Further, sentiment analysis [36,37] will be conducted to estimate a polarity, an emotion or a level of agreement by the user in relation to the message. Mapping the spread of sentiments in the social network can help to identify the main trends in opinion and possibly to characterize the “forces” (if any) acting behind these opinions.

## 6. Main Considerations and Challenges Ahead

From the very first known case of COVID-19 in China at the end of 2019, the world has been eager for good news. The promises of new vaccines are great, but many challenges are still to be tackled.

A clear, efficient and effective vaccination campaign must achieve the goal of directly vaccinating those at the highest risk for the severe outcome and protect them indirectly by vaccinating those who transmit the most.

Vaccination rollout speeds may differ depending on vaccine doses availability, logistical issues, but mostly on population vaccine attitudes and acceptance. A question is still open on this topic: once vaccines will be available for all, will we be able to use them?

For this purpose, communication plans play a central role and can make an important contribution to the achievement of this objective. Even if some technologies have never been used in a licensed vaccine before, the platforms were studied long before the pandemic, and this concept should be addressed when communicating with the public. Positively, as shown during the actual pandemic, public health professionals have also taken an active role in these conversations, becoming opinion leaders [38]. Nevertheless, strong attention must be paid to other self-calling experts or professionals who contribute to the spread of misinformation and/or unverified news.

The literature analyzed underlines how organizations and professionals in public health should roll out their sleeves and include the analysis of social media as an effective strategy to fight vaccine hesitancy [8]. Furthermore, social media is not only a platform for real-time surveillance of vaccine hesitancy and infectious diseases but also a useful communication tool for global health actors to identify which strategies are best suited to the different opinion groups in order to limit the spread of vaccine hesitancy and increase the levels of vaccine acceptance.

## Figures and Tables

**Table 1:** Main events related to days having a relative increase of tweets volume higher than 50%.

Date	ΔT	Main Events
20-10-2020	0.51	Rumors about vaccine in December
25-10-2020	0.55	Press conference about vaccine in December
9-11-2020	7.91	Pfizer’s vaccine announced
20-11-2020	0.56	Skeptical statement on vaccine by a well-known physician
2-12-2020	0.81	Vaccination plan presented by Minister of Health
26-12-2020	1.57	First doses of vaccine arrive in Italy
27-12-2020	1.71	First day of vaccination in Italy
24-01-2020	0.59	Controversy over a call for a pavilion for vaccinations

## Data Availability

Not applicable.
